# Adsorption of drugs on B_12_N_12_ and Al_12_N_12_ nanocages†

**DOI:** 10.1039/d4ra05586a

**Published:** 2024-10-08

**Authors:** Remya Geetha Sadasivan Nair, Arun Kumar Narayanan Nair, Shuyu Sun

**Affiliations:** a Physical Science and Engineering Division (PSE), Computational Transport Phenomena Laboratory, King Abdullah University of Science and Technology (KAUST) Thuwal 23955-6900 Saudi Arabia remya.nair@kaust.edu.sa arun.narayanannair@kaust.edu.sa shuyu.sun@kaust.edu.sa

## Abstract

The adsorption behavior of twelve drug molecules (5-fluorouracil, nitrosourea, pyrazinamide, sulfanilamide, ethionamide, 6-thioguanine, ciclopirox, 6-mercaptopurine, isoniazid, metformin, 4-aminopyridine, and cathinone) on B_12_N_12_ and Al_12_N_12_ nanocages was studied using density functional theory. In general, the drug molecules prefer to bind with the boron atom of the B_12_N_12_ nanocage and the aluminium atoms of the Al_12_N_12_ nanocage. However, a hydrogen atom is transferred from each of 5-fluorouracil, nitrosourea, 6-thioguanine, ciclopirox, and 6-mercaptopurine to the nitrogen atom of the Al_12_N_12_ nanocage. All the drug molecules are found to be chemisorbed on the B_12_N_12_ and Al_12_N_12_ nanocages. The adsorption energies of the drug/B_12_N_12_ system are linearly correlated with the molecular electrostatic potential minimum values of the drug molecules. The transfer of the hydrogen atom from the drug molecules to the nitrogen atom of the Al_12_N_12_ nanocage leads to relatively high adsorption energies. We observed significant changes in the reactivity parameters (*e.g.* electronic chemical potential) of the nanocages due to the chemisorption process. Overall, the QTAIM analysis indicates that the interactions between drug molecules and nanocages have a partial covalent character. Among the studied systems, the adsorption process was more spontaneous for the ciclopirox/Al_12_N_12_ system in water.

## Abstract 2

DFT studies were carried out to understand the adsorption mechanism of twelve drug molecules on the B_12_N_12_ and Al_12_N_12_ nanocages.

## Graphical abstract

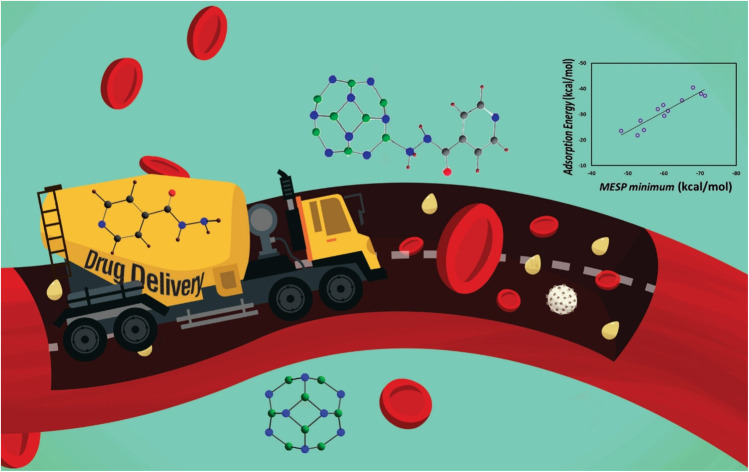

## 1 Introduction

Many drugs have been developed to treat various diseases and improve human health.^1–10^ For example, 5-fluorouracil, a pyrimidine containing drug, is widely used in the management of different types of cancers such as colon cancer and head and neck cancer.^1^ Nitrosoureas have long been of interest in the treatment of brain tumors and Hodgkin's disease.^2^ Pyrazinamide, ethionamide, and isoniazid play key roles in the treatment of tuberculosis.^3,4^ Sulfanilamide could be used in the treatment of vaginal infections.^5^ 6-Thioguanine and 6-mercaptopurine are important in the treatment of lymphoblastic leukemia.^6^ Ciclopirox is an ideal candidate for the treatment of superficial dermatophyte and yeast infections.^7^ Metformin can be used to lower blood glucose in non-insulin-dependent diabetic patients.^8^ 4-Aminopyridine is reported to be useful for managing the symptoms of multiple sclerosis.^9^ There has been interest in the therapeutic potential of cathinone as an antidepressant.^10^

There has been growing interest in developing materials for drug delivery and sensing applications.^11–34^ Density functional theory (DFT) was used to study the adsorption of drug molecules on nanosheets and nanotubes.^15–23^ DFT investigations revealed that the adsorption energy of isoniazid on B-doped carbon nanotubes was higher than that on the pristine carbon nanotubes.^15^ The adsorption of metformin on carbon nanotube was physisorption in nature, while that on Al- and Si-doped carbon nanotubes was chemisorption in nature.^16^ 5-Fluorouracil was physically adsorbed on the graphene oxide nanosheet.^17^ The adsorption process of 5-fluorouracil, 6-thioguanine, and 6-mercaptopurine on the boron nitride nanosheet was exothermic and occurred spontaneously.^18^ Nitrosourea was found to be physically adsorbed on the boron nitride nanosheet.^19^ The adsorption energy of 5-fluorouracil on Al-doped boron nitride nanotube was higher than that on the pristine boron nitride nanotube.^20^ The binding stability on a graphene flake decreased in the sequence 6-thioguanine > 6-mercaptopurine (thiol form) > 5-fluorouracil.^21^ 5-Fluorouracil was physically adsorbed to the wall of the carbon nanotube, while a chemisorption occurred between 5-fluorouracil and doped carbon nanotube.^22^ The adsorption of 5-fluorouracil on AlN-nanotube was physisorption in nature.^23^

There have also been studies on the adsorption of drug molecules on fullerene-like nanocages such as B_12_N_12_ and Al_12_N_12_ using DFT.^24–34^ The nanocage clusters of B_12_N_12_ were synthesized by Oku *et al.* in 2004.^35^ The AlN nanostructures have also been successfully synthesized.^36,37^ The Al_12_N_12_ nanocage was predicted to be the most stable among the Al_*n*_N_*n*_ (*n* = 2–41) nanocages.^38^ DFT investigations revealed that the B_12_N_12_ nanocage is a better sensor for 4-aminopyridine than the Al_12_N_12_ nanocage.^27^ 6-Mercaptopurine binds *via* the unsubstituted nitrogen atom of the imidazole ring to the B_12_N_12_ nanocage.^28^ The B_12_N_12_ nanocage could be used as a potential sensor for the detection of metformin.^29^ The adsorption of 6-thioguanine onto the B_12_N_12_ nanocage was a strong chemisorption in the gas phase as well as in water.^30^ Sulfanilamide preferred to bind with the boron atom of the B_12_N_12_ nanocage and the aluminium atom of the Al_12_N_12_ nanocage.^31^ The oxygen atom of the carbonyl group of ciclopirox bound to the boron atom of the B_12_N_12_ nanocage.^32^ The B_12_N_12_ nanocage could be a potential candidate as a drug carrier for isoniazid.^33^ A hydrogen atom was transferred from ciclopirox to the nitrogen atom of the Al_12_N_12_ nanocage.^34^ However, the adsorption properties of drug molecules like ethionamide on the B_12_N_12_ and Al_12_N_12_ nanocages have yet to be investigated.

In this work, the adsorption behavior of twelve drug molecules on the B_12_N_12_ and Al_12_N_12_ nanocages was studied using DFT. Typically, the molecular electrostatic potential (MESP) minimum (*V*_min_) points appear along the electron-rich regions (*e.g.*, π- and lone-pair regions).^39–42^ An interesting observation is that the adsorption energies of the drug/Bl_12_N_12_ system are linearly correlated with the MESP *V*_min_ values of the drug molecules. The present study may be helpful for the exploration of nanocages in drug delivery and sensing applications.

## 2 Computational details

**Fig. 1:**
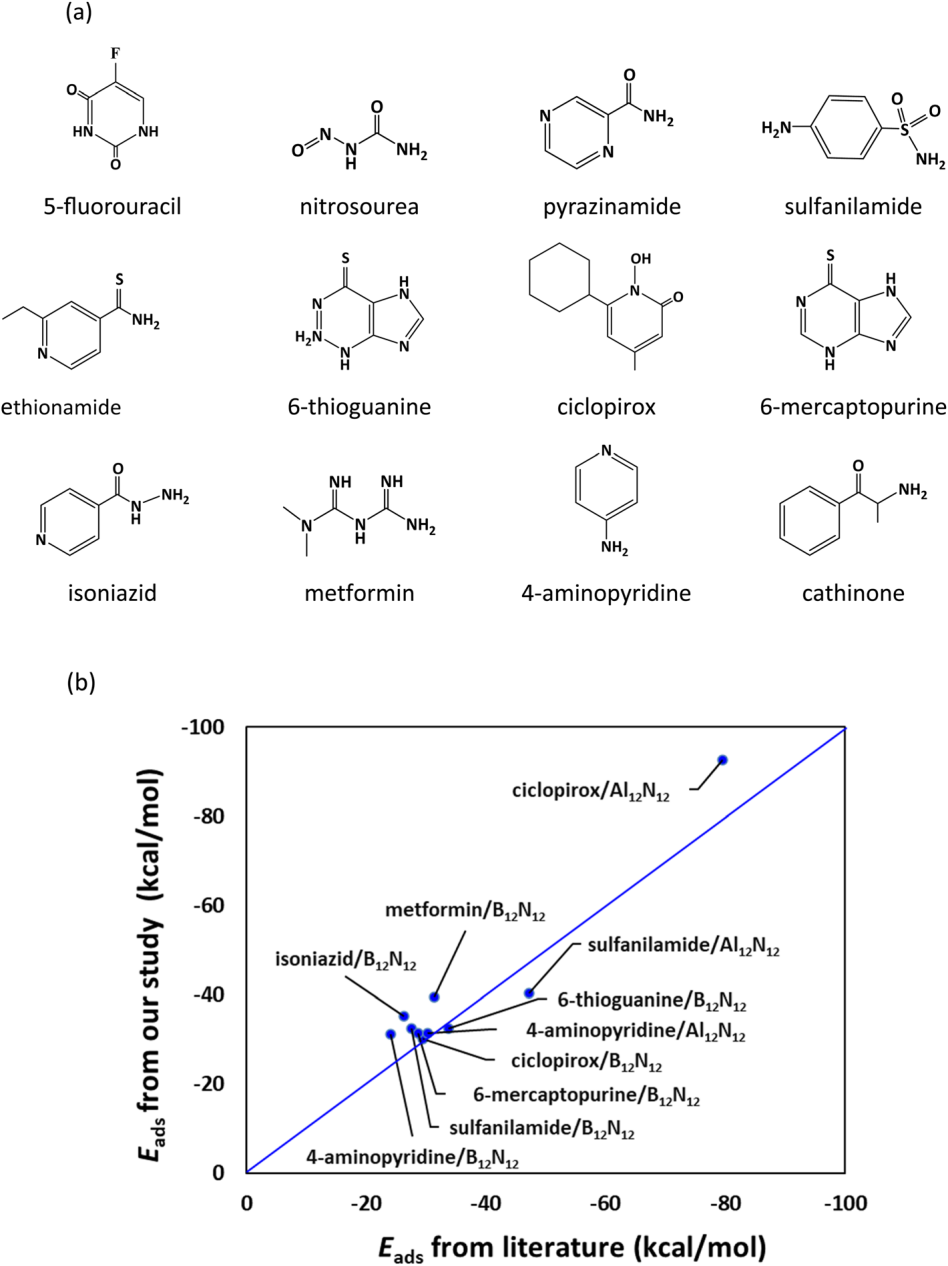
(a) Drug molecules examined in the present study. (b) Comparison of our results for adsorption energies with literature values.^27–34^

The adsorption of twelve drug molecules, namely, 5-fluorouracil, nitrosourea, pyrazinamide, sulfanilamide, ethionamide, 6-thioguanine, ciclopirox, 6-mercaptopurine, isoniazid, metformin, 4-aminopyridine, and cathinone onto the B_12_N_12_ and Al_12_N_12_ nanocages was studied using DFT. The chemical structures of the drug molecules examined in the present study are given in Fig. 1a. DFT computations were conducted using the Gaussian 16 program.^43^ All structures are optimized at the M062X/6-311G(d,p) level and confirmed as energy minima by frequency calculations.^44^ The MESP, *V*(*r*), is given as^39,40,42,45^1
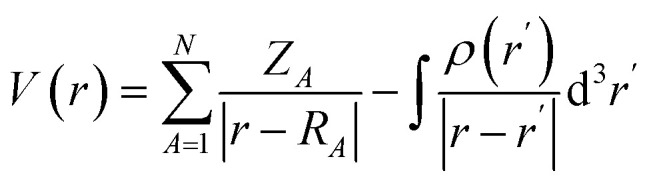
where *Z*_*A*_ is the nuclear charge of *A* positioned at *R*_*A*_ and *ρ*(*r*) is the electron density. *V*(*r*) is positive if the first term (nuclear contribution) in eqn (1) dominates and negative if the second term (electronic contribution) in eqn (1) dominates.

The adsorption energy of the drug molecule on the nanocage (*E*_ads_) is computed as follows:

2 *E*_ads_ = *E*_drug/nanocage_ − (*E*_nanocage_ + *E*_drug_) + *E*_BSSE_

where *E*_drug/nanocage_, *E*_nanocage_, and *E*_drug_ are the energy of the drug-adsorbed nanocage, the nanocage, and the drug molecule, respectively. *E*_BSSE_ denote the basis set superposition error (BSSE) correction obtained by the counterpoise method.^46^ M06-2X is a hybrid meta functional with 54% of exact Hartree–Fock (HF) exchange. It is a high-nonlocality functional with double the amount of nonlocal exchange (2X) and it also considers the dispersion forces.^44,47^ The M06-2X functional is parameterized for nonmetals and recommended for the study of noncovalent interactions, kinetics, and main-group thermochemistry. Fig. 1b shows a comparison of our computed *E*_ads_ values with the DFT results from the literature^27–34^ (see also Table S1, ESI†). Our computed *E*_ads_ values are in reasonable agreement with previous results.

The Gibbs free energy change (Δ*G*), the enthalpy change (Δ*H*), and the entropy change (Δ*S*) were estimated by the following equations:

3 Δ*G* = *G*_drug/nanocage_ − (*G*_nanocage_ + *G*_drug_)

4 Δ*H* = *H*_drug/nanocage_ − (*H*_nanocage_ + *H*_drug_)

5
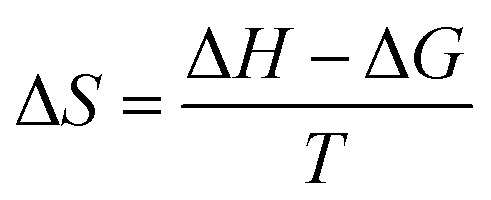
Here, *G*_drug/nanocage_, *G*_nanocage_, and *G*_drug_ are the free energy of the drug-adsorbed nanocage, the nanocage, and the drug molecule, respectively. *H*_drug/nanocage_, *H*_nanocage_, and *H*_drug_ are the enthalpy of the drug-adsorbed nanocage, the nanocage, and the drug molecule, respectively. *T* is the room temperature (*T* = 298.15 K).

The DFT reactivity indices were estimated by the following equations:^48^6
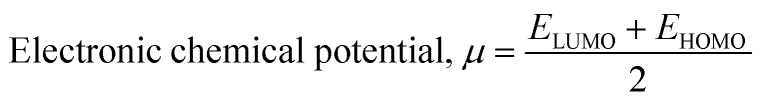
7
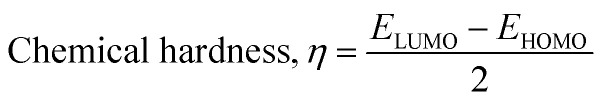
8
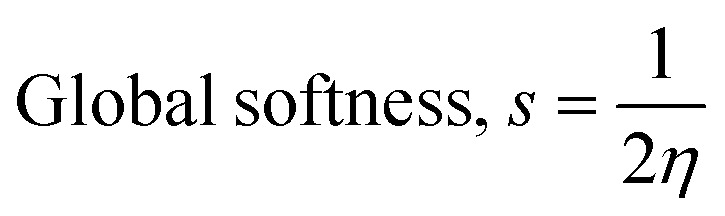
9
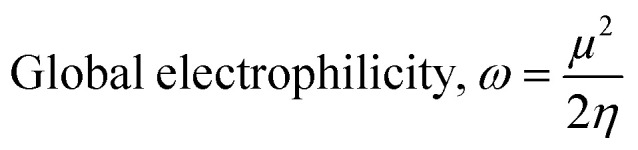
where *E*_HOMO_ denotes the energy of the highest occupied molecular orbital (HOMO) and *E*_LUMO_ denotes the energy of the lowest unoccupied molecular orbital (LUMO). The above DFT reactivity indices can play an important role in the understanding of chemical reactions.^39,42,48^

Bader's quantum theory of atoms in molecules (QTAIM) analyses^49^ were conducted at the M062X/6-31G(d,p) level using the Multiwfn software.^50^ The values of *ρ*_b_ and its Laplacian (∇^2^*ρ*_b_) and the total electron energy density (*H*_b_) and its components (the kinetic electron energy density (*G*_b_) and the potential electron energy density (*V*_b_)) at the bond critical point can provide insights into the nature of the atomic interactions.^51^ For example, ∇^2^*ρ*_b_ < 0 generally indicates covalent interactions. ∇^2^*ρ*_b_ > 0 and *H*_b_ > 0 indicate noncovalent interactions such as van der Waals and electrostatic interactions, while ∇^2^*ρ*_b_ > 0 and *H*_b_ < 0 indicate partially covalent interactions. In addition, −*G*_b_/*V*_b_ < 0.5, 0.5 < −*G*_b_/*V*_b_ < 1, and −*G*_b_/*V*_b_ > 1 indicate covalent, partially covalent and noncovalent interactions, respectively.^39,42,51^

## 3 Results and discussion

### 3.1 MESP

**Fig. 2:**
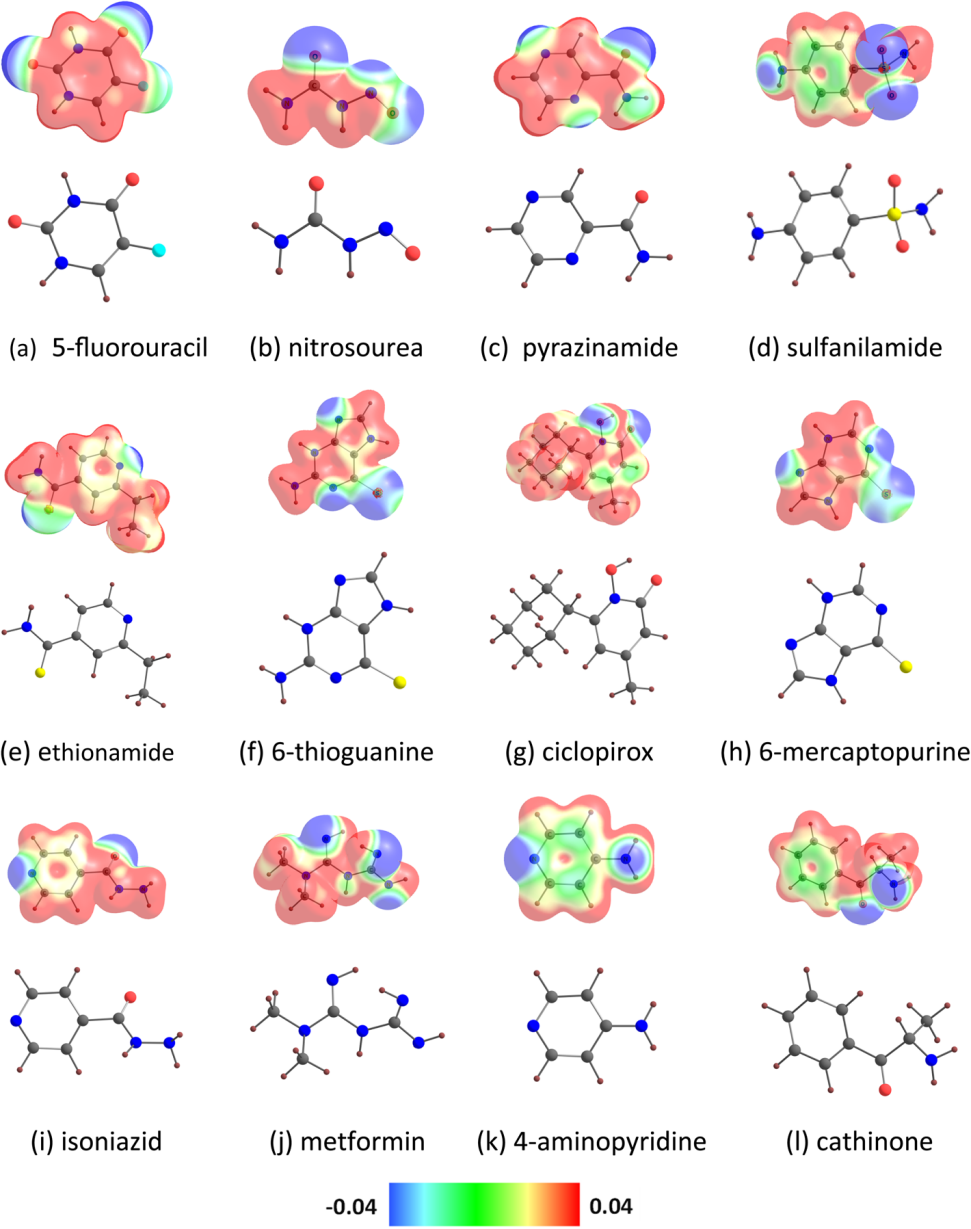
MESP mapped onto the 0.01 a.u. electron density isosurface of (a) 5-fluorouracil, (b) nitrosourea, (c) pyrazinamide, (d) sulfanilamide, (e) ethionamide, (f) 6-thioguanine, (g) ciclopirox, (h) 6-mercaptopurine, (i) isoniazid, (j) metformin, (k) 4-aminopyridine and (l) cathinone. Color code: gray-C, blue-N, yellow-S, maroon-H, cyan-F, red-O. Color code: blue −0.04 a.u. to red 0.04 a.u. Blue represents the most electron-rich region and red the most electron-poor region.

The MESP isosurfaces of the drug molecules are given in Fig. 2. The visual inspection of the MESP surfaces indicates that electron-rich regions (*e.g.*, blue regions) are present in the drug molecules. For example, the blue regions in the MESP maps of 5-fluorouracil are mainly located near the oxygen atoms and of cathinone are located near the nitrogen and oxygen atoms. The locations of the MESP *V*_min_ of the drug molecules are given in Fig. S1.† The MESP *V*_min_ of 5-fluorouracil resides near the oxygen atom (in the *ortho* position relative to the fluorine). The MESP *V*_min_ of nitrosourea, pyrazinamide, and ciclopirox is observed near the oxygen atom of the carbonyl group. The MESP *V*_min_ points of sulfanilamide are found near the two oxygen atoms. The MESP *V*_min_ of ethionamide and 4-aminopyridine is observed near the pyridinic nitrogen atom. The MESP *V*_min_ of 6-thioguanine and 6-mercaptopurine is found near the unsubstituted nitrogen atom of the pyrimidine ring. The MESP *V*_min_ of isoniazid is observed near the nitrogen atom of the terminal amino group. The MESP *V*_min_ points of metformin are found near the nitrogen atoms of the two imine groups. The MESP *V*_min_ of cathinone resides near its nitrogen atom. Furthermore, the MESP *V*_min_ values of the drug molecules (represented as *V*_min-X_) are given in Table 1. Here the *V*_min-X_ values are in the range of −48.19 (5-fluorouracil) to −71.35 kcal mol^−1^ (cathinone). A higher negative MESP *V*_min_ value indicates a more electron rich character of the drug molecule. The MESP *V*_min_ values of benzene-containing drug molecules follow the order: sulfanilamide < cathinone. The MESP *V*_min_ values of pyridine-containing drug molecules follow the order: ethionamide < ciclopirox < isoniazid < 4-aminopyridine. The MESP *V*_min_ value of 5-fluorouracil is lower than that of pyrazinamide (−53.53 kcal mol^−1^). The MESP *V*_min_ values of purine-containing drug molecules follow the order: 6-thioguanine < 6-mercaptopurine. The MESP *V*_min_ value of nitrosourea (−52.71 kcal mol^−1^) is lower than that of metformin (−68.02 kcal mol^−1^).

**Table 1:** MESP *V*_min-X_ (kcal mol^−1^) for the drug molecules

Drug	*V*_min-X_
5-Fluorouracil	−48.19
Nitrosourea	−52.71
Pyrazinamide	−53.53
Sulfanilamide	−54.47
Ethionamide	−58.30
6-Thioguanine	−59.80
Ciclopirox	−60.12
6-Mercaptopurine	−61.12
Isoniazid	−65.01
Metformin	−68.02
4-Aminopyridine	−70.28
Cathinone	−71.35

The B_12_N_12_ nanocage consists of six tetragonal and eight hexagonal rings^39,52^ (Fig. S2†). This nanocage has two distinct B–N bonds (two hexagonal rings shared the shorter B–N bond (1.44 Å), and a tetragonal ring and a hexagonal ring shared the longer B–N bond (1.48 Å)). A similar structure was found for the Al_12_N_12_ nanocage (see Fig. S2†). Here, the shorter Al–N bond length is 1.78 Å, and the longer one is 1.85 Å. The visual inspection of the MESP surfaces indicates that electron-rich regions (*e.g.*, blue regions) are situated close to the nitrogen atoms of the B_12_N_12_ and Al_12_N_12_ nanocages (see Fig. S2†). The values of the MESP *V*_min_ of the B_12_N_12_ and Al_12_N_12_ nanocages (denoted as *V*_min-C_) were calculated to be −20.77 and −49.07 kcal mol^−1^, respectively.^39^

### 3.2 Adsorption of drug molecules on the B_12_N_12_ nanocage

**Fig. 3:**
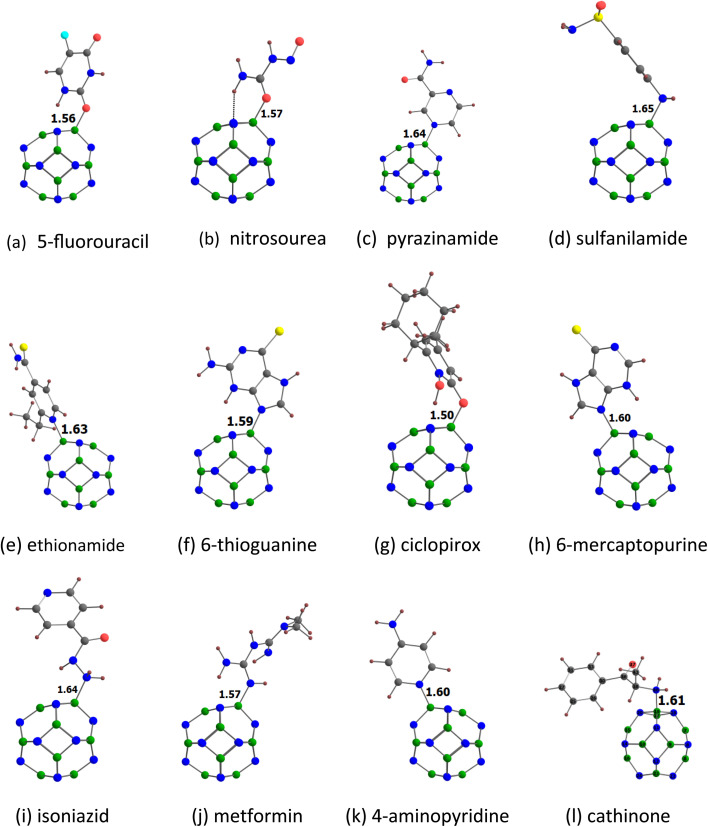
Optimized structures of drugs (a) 5-fluorouracil, (b) nitrosourea, (c) pyrazinamide, (d) sulfanilamide, (e) ethionamide, (f) 6-thioguanine, (g) ciclopirox, (h) 6-mercaptopurine, (i) isoniazid, (j) metformin, (k) 4-aminopyridine and (l) cathinone adsorbed on B_12_N_12_. The adsorption distances are given in Å. The color code is the same as in Fig. 2. In addition, B atom is denoted by green color.

**Fig. 4:**
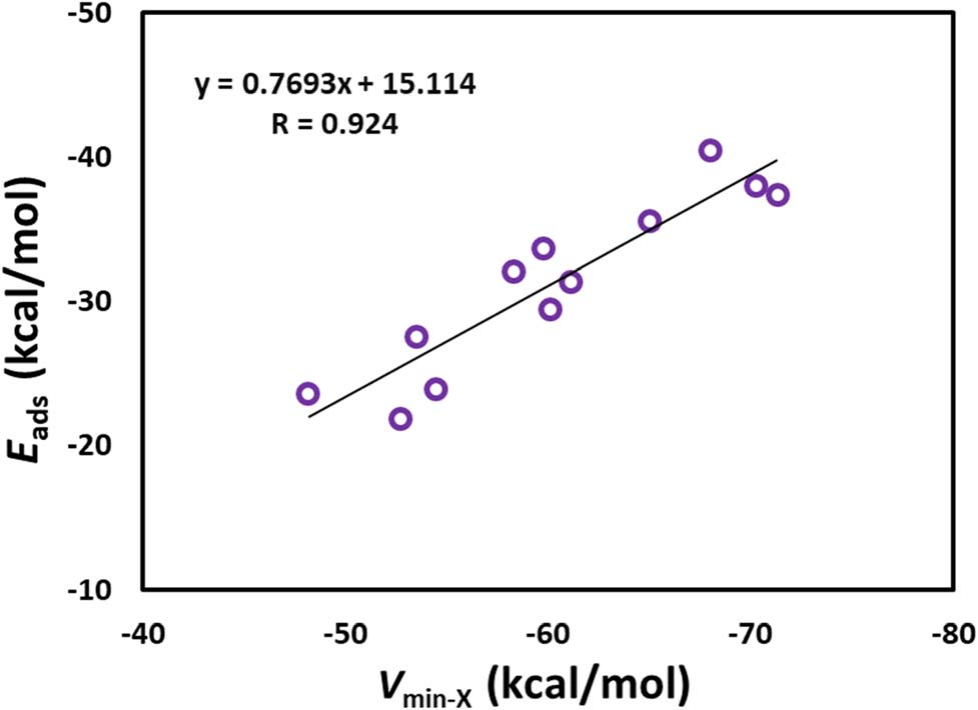
Correlation between *V*_min-X_ and *E*_ads_ for drug-adsorbed B_12_N_12_ nanocage.

Fig. 3 shows the optimized structures of the drug molecules adsorbed on the B_12_N_12_ nanocage. We see that all drug molecules prefer to bind with the boron atom of the B_12_N_12_ nanocage. 5-Fluorouracil binds *via* the oxygen atom (at the para position relative to the fluorine) to the B_12_N_12_ nanocage. Nitrosourea and ciclopirox bind *via* the oxygen atom of the carbonyl group. Pyrazinamide binds *via* the nitrogen atom (far from the amide group) of the pyrazine ring. Sulfanilamide binds *via* the nitrogen atom of the amino group attached to the benzene ring. Ethionamide and 4-aminopyridine bind *via* the pyridinic nitrogen atom. 6-Thioguanine and 6-mercaptopurine bind *via* the unsubstituted nitrogen atom of the imidazole ring. Isoniazid binds *via* the nitrogen atom of the terminal amino group. Metformin binds *via* the nitrogen atom of the imine group. Cathinone binds *via* its nitrogen atom to the B_12_N_12_ nanocage. Furthermore, all the drug molecules are found to be chemisorbed on the B_12_N_12_ nanocage. For example, the adsorption distances are in the range of 1.50 (ciclopirox) to 1.65 Å (sulfanilamide). This observation is also supported by the *E*_ads_ data (see Table 2) and other adsorption-induced structural changes (Table S2†). All these *E*_ads_ values are negative, and they are in the range of −21.85 (nitrosourea) to −40.50 kcal mol^−1^ (metformin). A higher negative value of *E*_ads_ generally indicates a stronger interaction between the drug molecule and the B_12_N_12_ nanocage. The *E*_ads_ values of benzene-containing drug molecules follow the order: sulfanilamide < cathinone. The *E*_ads_ values of pyridine-containing drug molecules follow the order: ciclopirox < ethionamide < isoniazid < 4-aminopyridine. The *E*_ads_ value of 5-fluorouracil (−23.59 kcal mol^−1^) is lower than that of pyrazinamide (−27.53 kcal mol^−1^). The *E*_ads_ values of purine-containing drug molecules follow the order: 6-mercaptopurine < 6-thioguanine. The *E*_ads_ value of nitrosourea is about two times lower than that of metformin. A key finding is that these *E*_ads_ values are well correlated with the MESP *V*_min_ values of the drug molecules, with a correlation coefficient of 0.924 (Fig. 4). This result reflects the stronger interactions between the drug molecules and the B_12_N_12_ nanocage as the MESP *V*_min_ values of the drug molecules become more negative. The angles of the hexagonal rings of the pristine B_12_N_12_ nanocage are about 125°. These angles at the adsorption sites decrease by about 9° due to the adsorption of drugs in all cases (see Table S2†).

**Table 2:** *E*_ads_, Δ*H*, Δ*S*, Δ*G*, MESP *V*_min-C′_, and Δ*V*_min-C_ for drug-adsorbed B_12_N_12_ nanocage. Δ*S* is given in kcal mol^−1^ K^−1^, other values are given in kcal mol^−1^

Drug	*E*_ads_	Δ*H*	Δ*S*	Δ*G*	*V*_min-C′_	Δ*V*_min-C_
5-Fluorouracil	−23.59	−25.78	−0.04	−14.33	−34.39	−13.62
Nitrosourea	−21.85	−24.19	−0.04	−12.93	−39.66	−18.89
Pyrazinamide	−27.53	−28.65	−0.04	−17.61	−41.29	−20.52
Sulfanilamide	−23.90	−25.19	−0.03	−14.84	−41.35	−20.58
Ethionamide	−32.09	−33.08	−0.04	−21.27	−36.58	−15.81
6-Thioguanine	−33.64	−35.35	−0.04	−23.85	−47.44	−26.67
Ciclopirox	−29.44	−33.61	−0.04	−21.58	−45.87	−25.10
6-Mercaptopurine	−31.35	−32.90	−0.04	−21.41	−48.19	−27.42
Isoniazid	−35.52	−36.98	−0.04	−25.61	−48.88	−28.11
Metformin	−40.50	−42.77	−0.04	−31.47	−49.39	−28.62
4-Aminopyridine	−38.01	−39.05	−0.03	−29.13	−40.79	−20.02
Cathinone	−37.33	−39.25	−0.04	−27.44	−36.40	−15.63

The enthalpy change (Δ*H*), the entropy change (Δ*S*), and the Gibbs free energy change (Δ*G*) (see eqn (3)–(5)) for the drug/B_12_N_12_ system are provided in Table 2. The negative values of Δ*H* in all systems indicate that the adsorption processes are exothermic in nature. The values of Δ*H* are in the range of −24.19 (nitrosourea/B_12_N_12_ system) to −42.77 kcal mol^−1^ (metformin/B_12_N_12_ system). The values of Δ*S* are negative (about −0.04 kcal mol^−1^ K^−1^ in all cases), indicating a decrease in entropy during the adsorption process. In all cases, the spontaneous nature of the adsorption processes may be deduced from the fact that the estimated values of Δ*G* are negative. The values of Δ*G* are in the range of −12.93 (nitrosourea/B_12_N_12_ system) to −31.47 kcal mol^−1^ (metformin/B_12_N_12_ system). Here the values of Δ*G* are less negative than those of Δ*H* due to the entropic effect.

The MESP isosurfaces of the drug-adsorbed B_12_N_12_ nanocage are shown in Fig. S3.† The visual inspection indicates major alterations in the MESP features of the isolated molecules due to the chemisorption process (see also Fig. 2 and S2†). For example, the blue region near the nitrogen atom of cathinone turns red in the presence of B_12_N_12_. The values of Δ*V*_min-C_ = *V*_min-C′_ − *V*_min-C_ (*V*_min-C′_ is the MESP *V*_min_ of the drug-adsorbed nanocage) are provided in Table 2. In all cases, the Δ*V*_min-C_ values are negative, implying that the B_12_N_12_ nanocage becomes electron-rich upon adsorption of the drug molecules. Here the values of Δ*V*_min-C_ are in the range of −13.62 (5-fluorouracil/B_12_N_12_ system) to −28.62 kcal mol^−1^ (metformin/B_12_N_12_ system).

The adsorption of the drug molecules onto the nanocage may have an impact on the DFT reactivity indices *μ*, *η*, *s*, and *ω* (see eqn (6)–(9)). The electrophilicity index *ω* incorporates the tendency of a system to accept additional electronic charge (described by *μ*^2^) and the resistance of a system to change its electronic configuration (described by *η*). Thus, a good electrophile can be identified by a high *μ* value and a low *η* value. The values of *μ*, *η*, *s*, and *ω* for the pristine B_12_N_12_ nanocage were −4.73 eV, 4.72 eV, 0.11 eV^−1^ and 2.37 eV, respectively.^39^ The change in the DFT reactivity indices, for instance, Δ*μ* was estimated by taking the difference between the *μ* of the drug-adsorbed nanocage and the *μ* of the pristine nanocage. The values of Δ*μ*, Δ*η*, Δ*s*, and Δ*ω* are given in Table 3. In all cases, we observe significant changes in *μ*, *η*, *s*, and *ω* due to the chemisorption process. For instance, the values of Δ*μ* and Δ*η* for the 5-fluorouracil/B_12_N_12_ system are 7.71 and −23.64% respectively.

**Table 3:** The reactivity indices, *μ*, *η*, *s* and *ω* for drug-adsorbed B_12_N_12_ nanocage. The values of *μ*, *η*, and *ω* are given in eV; the values of *s* in (eV)^−1^; Δ*μ*, Δ*η*, Δ*s* and Δ*ω* in %

Drug	*μ*	Δ*μ*	*η*	Δ*η*	*s*	Δ*s*	*ω*	Δ*ω*
5-Fluorouracil	−5.09	7.71	3.60	−23.64	0.14	26.12	3.60	51.93
Nitrosourea	−5.21	10.15	3.31	−29.90	0.15	37.37	4.10	73.08
Pyrazinamide	−5.53	16.97	3.02	−35.98	0.17	50.42	5.06	113.69
Sulfanilamide	−4.84	2.30	3.85	−18.40	0.13	18.02	3.04	28.26
Ethionamide	−5.28	11.53	2.98	−36.93	0.17	52.68	4.67	97.22
6-Thioguanine	−4.66	−1.46	3.07	−35.03	0.16	48.21	3.54	49.43
Ciclopirox	−4.81	1.64	3.50	−25.92	0.14	30.00	3.31	39.46
6-Mercaptopurine	−5.01	6.01	3.00	−36.35	0.17	51.30	4.18	76.57
Isoniazid	−5.22	10.35	3.66	−22.49	0.14	24.25	3.72	57.12
Metformin	−4.18	−11.56	4.07	−13.67	0.12	11.56	2.15	−9.39
4-Aminopyridine	−4.48	−5.20	3.68	−21.94	0.14	23.38	2.73	15.13
Cathinone	−4.90	3.68	3.60	−23.70	0.14	26.22	3.34	40.89

The results from the QTAIM analyses of the drug-adsorbed B_12_N_12_ nanocage are given in Fig. S4† and Table 4. For all systems, the values of *ρ*_b_ are in the range of 0.108 (nitrosourea/B_12_N_12_ system) to 0.135 au (metformin/B_12_N_12_ system) and the values of ∇^2^*ρ*_b_ are positive (see Table 4). It can be seen that all the values of *H*_b_ are negative and 0.5 < −*G*_b_/*V*_b_ < 1. These results imply the presence of partial covalent interactions between the drug molecules and the B_12_N_12_ nanocage.

**Table 4:** The QTAIM parameters (a.u.) for the drug-adsorbed B_12_N_12_ nanocage

Drug	*ρ*_b_	∇^2^*ρ*_b_	*H*_b_	*G*_b_	*V*_b_	−*G*_b_/*V*_b_
5-Fluorouracil	0.111	0.496	−0.060	0.185	−0.245	0.753
Nitrosourea	0.108	0.489	−0.059	0.181	−0.240	0.755
Pyrazinamide	0.115	0.306	−0.079	0.156	−0.235	0.663
Sulfanilamide	0.114	0.277	−0.080	0.149	−0.229	0.651
Ethionamide	0.119	0.289	−0.085	0.157	−0.242	0.649
6-Thioguanine	0.125	0.364	−0.088	0.179	−0.268	0.670
Ciclopirox	0.133	0.579	−0.082	0.227	−0.309	0.734
6-Mercaptopurine	0.123	0.364	−0.086	0.177	−0.262	0.674
Isoniazid	0.116	0.301	−0.081	0.156	−0.237	0.659
Metformin	0.135	0.353	−0.100	0.188	−0.288	0.654
4-Aminopyridine	0.127	0.317	−0.092	0.171	−0.264	0.650
Cathinone	0.126	0.307	−0.092	0.169	−0.260	0.647

### 3.3 Adsorption of drug molecules on the Al_12_N_12_ nanocage

**Fig. 5:**
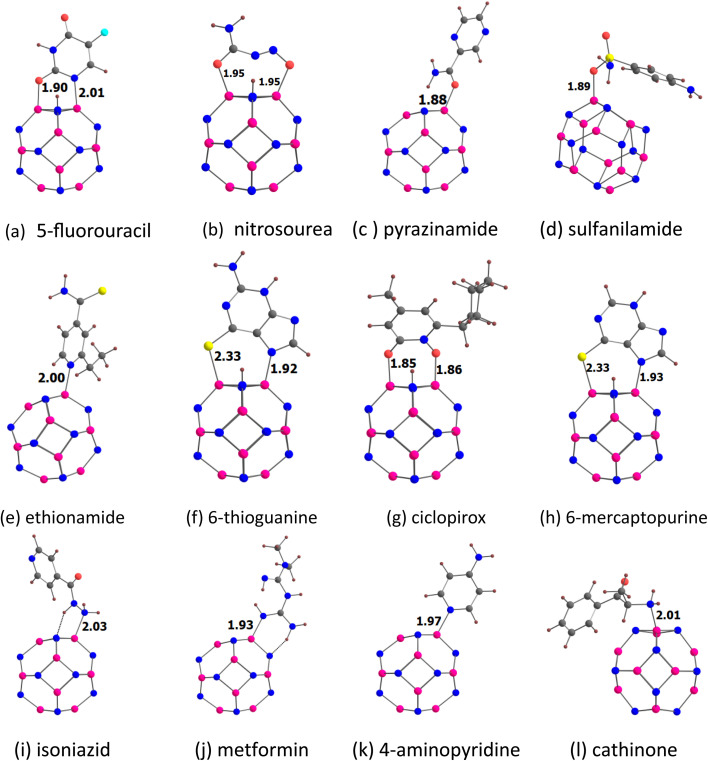
Optimized structures of drugs (a) 5-fluorouracil, (b) nitrosourea, (c) pyrazinamide, (d) sulfanilamide, (e) ethionamide, (f) 6-thioguanine, (g) ciclopirox, (h) 6-mercaptopurine, (i) isoniazid, (j) metformin, (k) 4-aminopyridine and (l) cathinone adsorbed on Al_12_N_12_. The adsorption distances are given in Å. The color code is the same as in Fig. 2. In addition, Al atom is denoted by purple color.

Fig. 5 shows the optimized structures of the drug molecules adsorbed on the Al_12_N_12_ nanocage. In general, the drug molecules prefer to bind with the aluminium atoms of the Al_12_N_12_ nanocage. 5-Fluorouracil binds, for example, *via* the oxygen atom (at the para position relative to the fluorine) to the Al_12_N_12_ nanocage. Nitrosourea and ciclopirox bind, for example, *via* the oxygen atom of the carbonyl group. Pyrazinamide and sulfanilamide bind *via* the oxygen atom. Ethionamide and 4-aminopyridine bind *via* the pyridinic nitrogen atom. 6-Thioguanine and 6-mercaptopurine bind, for example, *via* the nitrogen atom of the imidazole ring. Isoniazid binds *via* the nitrogen atom of the terminal amino group. Metformin binds *via* the nitrogen atom of the imine group. Cathinone binds *via* its nitrogen atom to the Al_12_N_12_ nanocage. However, a hydrogen atom is transferred from each of 5-fluorouracil, nitrosourea, 6-thioguanine, ciclopirox, and 6-mercaptopurine to the nitrogen atom of the Al_12_N_12_ nanocage. Similar transfer of the hydrogen atom from the drug molecules to the nitrogen atom of the nanocage has been reported previously.^34,53–61^ Furthermore, all the drug molecules are found to be chemisorbed on the A_12_N_12_ nanocage. For example, the adsorption distances are in the range of 1.85 (ciclopirox) to 2.03 Å (isoniazid). This observation is also supported by the *E*_ads_ data (see Table 5) and other adsorption-induced structural changes (Table S3†). All these *E*_ads_ values are negative, and they are in the range of −39.31 (ethionamide) to −84.81 kcal mol^−1^ (ciclopirox). A higher negative value of *E*_ads_ generally indicates a stronger interaction between the drug molecule and the Al_12_N_12_ nanocage. The transfer of the hydrogen atom from 5-fluorouracil, nitrosourea, 6-thioguanine, ciclopirox, and 6-mercaptopurine to the nitrogen atom of the A_12_N_12_ nanocage leads to relatively high *E*_ads_ values. The *E*_ads_ values of benzene-containing drug molecules follow the order: sulfanilamide < cathinone. The *E*_ads_ values of pyridine-containing drug molecules follow the order: ethionamide < 4-aminopyridine < isoniazid < ciclopirox. The *E*_ads_ value of 5-fluorouracil (−69.62 kcal mol^−1^) is higher than that of pyrazinamide (−42.27 kcal mol^−1^). The *E*_ads_ values of purine-containing drug molecules 6-mercaptopurine and 6-thioguanine are close to each other. The *E*_ads_ value of nitrosourea (−73.16 kcal mol^−1^) is higher than that of metformin (−50.71 kcal mol^−1^). These *E*_ads_ values are not correlated with the MESP *V*_min_ values of the drug molecules (Fig. S5†). The angles of the hexagonal rings of the pristine Al_12_N_12_ nanocage are about 125°. These angles at the adsorption sites decrease by at least 4° due to the adsorption of drugs (see Table S3†).

**Table 5:** *E*_ads_, Δ*H*, Δ*G*, Δ*S*, MESP *V*_min-C′_, and Δ*V*_min-C_ for drug-adsorbed Al_12_N_12_ nanocage. Δ*S* is given in kcal mol^−1^ K^−1^, other values are given in kcal mol^−1^

Drug	*E*_ads_	Δ*H*	Δ*G*	Δ*S*	*V*_min-C′_	Δ*V*_min-C_
5-Fluorouracil	−69.62	−75.76	−63.18	−0.04	−58.23	−9.16
Nitrosourea	−73.16	−81.63	−68.62	−0.04	−64.51	−15.44
Pyrazinamide	−42.27	−45.63	−34.38	−0.04	−66.39	−17.32
Sulfanilamide	−40.38	−46.48	−33.20	−0.04	−66.20	−17.13
Ethionamide	−39.31	−40.79	−29.64	−0.04	−62.00	−12.93
6-Thioguanine	−82.46	−88.02	−75.78	−0.04	−66.83	−17.76
Ciclopirox	−84.81	−91.39	−78.76	−0.04	−64.19	−15.12
6-Mercaptopurine	−82.31	−87.77	−75.45	−0.04	−63.19	−14.12
Isoniazid	−44.08	−46.41	−35.95	−0.04	−57.04	−7.97
Metformin	−50.71	−54.12	−43.22	−0.04	−67.83	−18.76
4-Aminopyridine	−42.69	−44.03	−34.48	−0.03	−69.09	−20.02
Cathinone	−42.98	−46.46	−34.72	−0.04	−64.95	−15.88

The Δ*H*, Δ*S*, and Δ*G* (see eqn (3)–(5)) for the drug/Al_12_N_12_ system are provided in Table 5. The negative values of Δ*H* in all systems indicate that the adsorption processes are exothermic in nature. The values of Δ*H* are in the range of −40.79 (ethionamide/Al_12_N_12_ system) to −91.39 kcal mol^−1^ (ciclopirox/Al_12_N_12_ system). The values of Δ*S* are negative (about −0.04 kcal mol^−1^ K^−1^ in all cases), indicating a decrease in entropy during the adsorption process. In all cases, the spontaneous nature of the adsorption processes may be deduced from the fact that the estimated values of Δ*G* are negative. The values of Δ*G* are in the range of −29.64 (ethionamide/Al_12_N_12_ system) to −78.76 kcal mol^−1^ (ciclopirox/Al_12_N_12_ system). Here also the values of Δ*G* are less negative than those of Δ*H* due to the entropic effect.

The MESP isosurfaces of the drug-adsorbed Al_12_N_12_ nanocage are shown in Fig. S6.† The visual inspection indicates major alterations in the MESP features of the isolated molecules due to the chemisorption process (see also Fig. 2 and S2†). For example, the blue region near the nitrogen atom of the Al_12_N_12_ nanocage turns red due to the transfer of the hydrogen atom from 5-fluorouracil. In all cases, the Δ*V*_min-C_ values are negative, implying that the Al_12_N_12_ nanocage becomes electron-rich upon adsorption of the drug molecules (see Table 5). The values of Δ*V*_min-C_ are in the range of −7.97 (isoniazid/Al_12_N_12_ system) to −20.02 kcal mol^−1^ (4-aminopyridine/Al_12_N_12_ system).

The values of *μ*, *η*, *s*, and *ω* for the pristine Al_12_N_12_ nanocage were −4.86 eV, 3.16 eV, 0.16 eV^−1^ and 3.74 eV, respectively.^39^ The values of Δ*μ*, Δ*η*, Δ*s*, and Δ*ω* of the drug/Al_12_N_12_ nanocage system are given in Table 6. In all cases, we observe significant changes in *μ*, *η*, *s*, and *ω* due to the chemisorption process. For instance, the values of Δ*μ* and Δ*η* for the 5-fluorouracil/Al_12_N_12_ system are −4.63 and −3.16% respectively. Overall, the changes in all these reactivity indices of the drug/Al_12_N_12_ system are lower when compared to the drug/B_12_N_12_ system (see Table 3).

**Table 6:** The reactivity indices, *μ*, *η*, *s* and *ω* for drug-adsorbed Al_12_N_12_ nanocage. The values of *μ*, *η*, and *ω* are given in eV; the values of *s* in (eV)^−1^; Δ*μ*, Δ*η*, Δ*s* and Δ*ω* in %

Drug	*μ*	Δ*μ* (%)	*η*	Δ*η* (%)	*s*	Δ*s* (%)	*ω*	Δ*ω* (%)
5-Fluorouracil	−4.63	−4.63	3.06	−3.16	0.16	2.12	3.51	−6.15
Nitrosourea	−4.49	−7.71	2.94	−7.04	0.17	6.38	3.42	−8.44
Pyrazinamide	−4.67	−3.85	2.77	−12.37	0.18	12.86	3.94	5.43
Sulfanilamide	−4.47	−8.02	3.05	−3.64	0.16	2.63	3.28	−12.26
Ethionamide	−4.78	−1.55	2.71	−14.35	0.18	15.47	4.23	13.08
6-Thioguanine	−4.34	−10.70	2.99	−5.53	0.17	4.68	3.15	−15.66
Ciclopirox	−4.26	−12.27	3.05	−3.47	0.16	2.44	2.98	−20.33
6-Mercaptopurine	−4.58	−5.72	2.86	−9.49	0.17	9.26	3.67	−1.87
Isoniazid	−4.68	−3.76	3.15	−0.28	0.16	−0.83	3.47	−7.19
Metformin	−4.22	−13.17	3.05	−3.38	0.16	2.35	2.92	−22.02
4-Aminopyridine	−4.16	−14.48	3.10	−1.87	0.16	0.78	2.79	−25.53
Cathinone	−4.38	−9.98	3.05	−3.64	0.16	2.63	3.14	−15.96

The results from the QTAIM analyses of the drug-adsorbed Al_12_N_12_ nanocage are given in Fig. S7† and Table 7. For all systems, the values of *ρ*_b_ are in the range of 0.052 (6-mercaptopurine/Al_12_N_12_ system) to 0.068 au (6-thioguanine/Al_12_N_12_ system) and the values of ∇^2^*ρ*_b_ are positive (see Table 7). Overall, the values of *H*_b_ are negative and 0.5 < −*G*_b_/*V*_b_ < 1, suggesting the presence of partial covalent interactions between the drug molecules and the Al_12_N_12_ nanocage. However, the values of *H*_b_ are positive and −*G*_b_/*V*_b_ > 1 for the 5-fluorouracil/Al_12_N_12_ (Al–O bond), nitrosourea/Al_12_N_12_, pyrazinamide/Al_12_N_12_, sulfanilamide/Al_12_N_12_, and ciclopirox/Al_12_N_12_ systems. These results imply the presence of noncovalent interactions between these drug molecules and the Al_12_N_12_ nanocage.

**Table 7:** The QTAIM parameters (a.u.) for the drug-adsorbed Al_12_N_12_ nanocage

Drug	*ρ*_b_	∇^2^*ρ*_b_	*H*_b_	*G*_b_	*V*_b_	−*G*_b_/*V*_b_
5-Fluorouracil (Al–O)	0.061	0.419	0.004	0.101	−0.097	1.042
5-Fluorouracil (Al–N)	0.057	0.310	−0.002	0.080	−0.082	0.970
Nitrosourea (Al–OC)	0.054	0.347	0.003	0.083	−0.080	1.044
Nitrosourea (Al–ON)	0.056	0.338	0.001	0.083	−0.082	1.016
Pyrazinamide	0.062	0.457	0.007	0.108	−0.101	1.067
Sulfanilamide	0.058	0.424	0.007	0.099	−0.093	1.071
Ethionamide	0.057	0.321	−0.001	0.082	−0.083	0.985
6-Thioguanine (Al–S)	0.053	0.159	−0.013	0.053	−0.066	0.801
6-Thioguanine (Al–N)	0.068	0.411	−0.002	0.105	−0.107	0.980
Ciclopirox (Al–ON)	0.067	0.483	0.004	0.117	−0.113	1.037
Ciclopirox (Al–OC)	0.066	0.490	0.005	0.117	−0.112	1.048
6-Mercaptopurine (Al–S)	0.052	0.157	−0.013	0.052	−0.065	0.802
6-Mercaptopurine (Al–N)	0.067	0.405	−0.002	0.103	−0.105	0.982
Isoniazid	0.055	0.303	−0.001	0.077	−0.078	0.987
Metformin	0.066	0.400	−0.002	0.102	−0.103	0.984
4-Aminopyridine	0.061	0.361	−0.001	0.091	−0.093	0.986
Cathinone	0.057	0.318	−0.001	0.081	−0.082	0.983

### 3.4 Recovery time

A shorter recovery time (*τ*) is often required for the reusability of a sensor material (*e.g.* B_12_N_12_).^62,63^ The recovery time was calculated using the following equation:

10 *τ* = *ϑ*^−1^ exp(−*E*_ads_/(*k*_B_*T*))

where *ϑ* is the attempt frequency (10^18^ Hz) and *k*_B_ is the Boltzmann constant. The computed recovery time is listed in Table S4.† We find that *τ* is shortest for the nitrosourea/B_12_N_12_ system (∼0.01 s). The high adsorption energy of 5-fluorouracil, nitrosourea, 6-thioguanine, ciclopirox, and 6-mercaptopurine on the Al_12_N_12_ nanocage gives rise to a long recovery time.

### 3.5 Solvent effects

We investigated the effect of water, the most important biological solvent, on the interaction between the drug molecules and the nanocages. The M062X/6-311G(d,p) level energetics was corrected for solvation effects using the self-consistent reaction field method SMD.^64^ The solvent-corrected Gibbs free energy (Δ*G*_W_) was calculated by adding the solvent-phase single-point energy with the gas-phase Gibbs free energy correction. The Δ*G*_W_ values for the drug/B_12_N_12_ complex are provided in Table 8. The values of Δ*G*_W_ are in the range of −23.80 (5-fluorouracil/B_12_N_12_ complex) to −43.84 kcal mol^−1^ (metformin/B_12_N_12_ complex). A more negative Δ*G*_W_ indicates that the adsorption is more exergonic for the metformin/B_12_N_12_ complex in water.

**Table 8:** Δ*G*_W_ (kcal mol^−1^) for drug-adsorbed B_12_N_12_ and Al_12_N_12_ nanocages

Drug	Δ*G*_W_	
	Drug/B_12_N_12_	Drug/Al_12_N_12_
5-Fluorouracil	−23.80	−55.20
Nitrosourea	−24.64	−62.98
Pyrazinamide	−29.34	−30.11
Sulfanilamide	−28.75	−25.77
Ethionamide	−31.93	−26.56
6-Thioguanine	−31.03	−67.86
Ciclopirox	−33.54	−73.93
6-Mercaptopurine	−29.14	−66.94
Isoniazid	−33.77	−28.76
Metformin	−43.84	−40.72
4-Aminopyridine	−41.28	−33.10
Cathinone	−39.91	−33.51

The Δ*G*_W_ for the drug/Al_12_N_12_ complex is also provided in Table 8. Here the values of Δ*G*_W_ are in the range of −25.77 (sulfanilamide/Al_12_N_12_ complex) to −73.93 kcal mol^−1^ (ciclopirox/Al_12_N_12_ complex). A more negative Δ*G*_W_ indicates that the adsorption is more spontaneous for the ciclopirox/Al_12_N_12_ complex in water. This is possibly due to the presence of the –OH group in ciclopirox.

The MESP is a real physical property which can be obtained by computational method or experimentally by X-ray diffraction technique.^41^ The MESP *V*_min_ value would qualify as a good parameter for quantifying the strength of, for example, a lone pair.^41^ Typically, the electron-rich lone-pair regions of the drug molecules interact with the electron-deficient boron or aluminium atoms of the B_12_N_12_ and Al_12_N_12_ nanocages. We observed a linear correlation between the *E*_ads_ values of the drug-adsorbed B_12_N_12_ nanocage and the MESP *V*_min_ values of the drugs. This enables one to predict the adsorption energy once the MESP features of the drug molecules are known. Similar correlations were found for the lone pair-π interactions.^41^ However, the *E*_ads_ values of the drug-adsorbed Al_12_N_12_ nanocage were not correlated with the MESP *V*_min_ values of the drug molecules. Also, a hydrogen atom was transferred from each of 5-fluorouracil, nitrosourea, 6-thioguanine, ciclopirox, and 6-mercaptopurine to the nitrogen atom of the Al_12_N_12_ nanocage. These results may be attributed to the fact that the Al_12_N_12_ nanocage is more electron-rich compared to the B_12_N_12_ nanocage (MESP *V*_min_ of B_12_N_12_ and Al_12_N_12_ nanocages are −20.77 and −49.07 kcal mol^−1^, respectively). More negative electrostatic potentials at nuclei (EPN) values indicate greater electron densities in a molecular region.^65^ For the B_12_N_12_ nanocage, we estimated the EPN values at B and N to be −11.37 and −18.39 au, respectively. For the Al_12_N_12_ nanocage, the EPN values at Al and N are −44.55 and −18.45 au, respectively. Also, the Al_12_N_12_ nanocage displayed a relatively high surface area.^42^ The B–N bond lengths in the B_12_N_12_ nanocage were in the range of 1.44 to 1.48 Å and the Al–N bond lengths in the Al_12_N_12_ nanocage were in the range of 1.78 to 1.85 Å.^42^

All the drug molecules investigated in this study were found to be chemisorbed on the B_12_N_12_ and Al_12_N_12_ nanocages. In contrast, for example, the adsorption of 5-fluorouracil on AlN-nanotube^23^ and nitrosourea on BN-nanosheet^19^ were physisorption in nature. Structural defects in the nanocages or changes in the surface chemical environment^66,67^ may also affect the adsorption properties of drugs. We will study these effects in a future publication.

## 4 Conclusions

DFT studies were conducted to understand the adsorption mechanism of twelve drug molecules (5-fluorouracil, nitrosourea, pyrazinamide, sulfanilamide, ethionamide, 6-thioguanine, ciclopirox, 6-mercaptopurine, isoniazid, metformin, 4-aminopyridine, and cathinone) on the B_12_N_12_ and Al_12_N_12_ nanocages. In general, the drug molecules prefer to bind with the boron atom of the B_12_N_12_ nanocage and the aluminium atoms of the Al_12_N_12_ nanocage. However, a hydrogen atom is transferred from each of 5-fluorouracil, nitrosourea, 6-thioguanine, ciclopirox, and 6-mercaptopurine to the nitrogen atom of the Al_12_N_12_ nanocage. All the drug molecules were found to be chemisorbed on the B_12_N_12_ and Al_12_B_12_ nanocages. The adsorption distances are in the range of 1.50 (ciclopirox/B_12_N_12_ system) to 2.03 Å (isoniazid/Al_12_N_12_ system). All the *E*_ads_ values were negative, indicating the exothermic nature of the adsorption process. The *E*_ads_ values are in the range of −21.85 (nitrosourea/B_12_N_12_ system) to −84.81 kcal mol^−1^ (ciclopirox/Al_12_N_12_ system). A key finding is that the *E*_ads_ values of the drug/Bl_12_N_12_ system are linearly correlated with the MESP *V*_min_ values of the drug molecules. The transfer of the hydrogen atom from the drug molecules to the nitrogen atom of the A_12_N_12_ nanocage leads to relatively high *E*_ads_ values.

In all cases, the Δ*V*_min-C_ values are negative, implying that the B_12_N_12_ and Al_12_N_12_ nanocages become electron-rich upon adsorption of the drug molecules. We found significant changes in the reactivity parameters such as *μ* and *η* of the nanocages due to the chemisorption process. In general, the QTAIM results indicate the presence of partial covalent interactions between the drug molecules and the nanocages. However, the QTAIM results indicate the presence of noncovalent interactions for the 5-fluorouracil/Al_12_N_12_, nitrosourea/Al_12_N_12_, pyrazinamide/Al_12_N_12_, sulfanilamide/Al_12_N_12_, and ciclopirox/Al_12_N_12_ systems. We also investigated the effect of water on the interaction between the drug molecules and the nanocages. A more negative Δ*G*_W_ indicates that the adsorption is more exergonic for the ciclopirox/Al_12_N_12_ complex in water.

## Data availability

The data supporting this article have been included as part of the ESI.†

## Conflicts of interest

There are no conflicts to declare.

## Supplementary Material

RA-014-D4RA05586A-s001
