# Supplementary material for: Adsorption of drugs on B12N12 and Al12N12 nanocages
Source: RSC Adv. 2024 Oct 8;14(43):31756–67. doi: 10.1039/d4ra05586a (PMC11459447; doi:10.1039/d4ra05586a)
Supplement: RA-014-D4RA05586A-s001 [file RA-014-D4RA05586A-s001.pdf]

Supplementary Information (SI) for RSC Advances.
This journal is © The Royal Society of Chemistry 2024

Supporting Information

Adsorption of Drugs on $B_{12}N_{12}$ and $Al_{12}N_{12}$ nanocages

Remya Geetha Sadasivan Nair*, Arun Kumar Narayanan Nair*, and Shuyu Sun*

Physical Science and Engineering Division (PSE), Computational Transport Phenomena Laboratory, King Abdullah University of Science and Technology (KAUST), Thuwal, 23955-6900, Saudi Arabia.

* To whom correspondence should be addressed,

email: remya.nair@kaust.edu.sa; arun.narayanannair@kaust.edu.sa; shuyu.sun@kaust.edu.sa

Table S1. Comparison of our computed E_{ads} values with the DFT results from the literature. E_{ads} values are given in kcal/mol.

Drug/nanocage	E_{ads} (literature)	E_{ads} (our work)	% difference
sulfanilamide/ $B_{12}N_{12}$ [S1]	-27.53	-32.50	-15.3
sulfanilamide/ $Al_{12}N_{12}$ [S2]	-47.17	-40.38	16.8
6-thioguanine/ $B_{12}N_{12}$ [S2]	-33.64	-32.48	3.6
ciclopirox/ $B_{12}N_{12}$ [S3]	-29.44	-30.07	-2.1
4-amino pyridine/ $B_{12}N_{12}$[S4]	-24.05	-31.13	-22.7
4-amino pyridine/ $Al_{12}N_{12}$ [S4]	-30.29	-31.36	-3.4
ciclopirox/ $Al_{12}N_{12}$ [S5]	-79.56	-92.65	-14.1
metformin/ $B_{12}N_{12}$ [S6]	-31.36	-39.40	-20.4
isoniazid/ $B_{12}N_{12}$ [S7]	-26.32	-35.29	-25.4
6-mercaptopurine/ $B_{12}N_{12}$ [S8]	-28.60	-31.35	-8.8

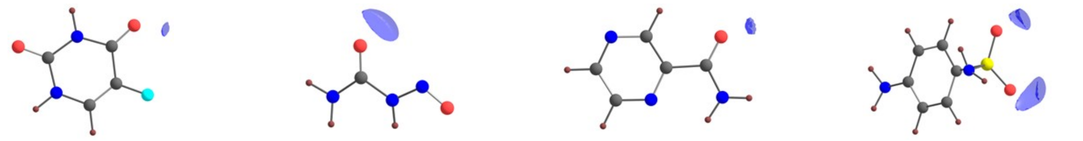

(a) 5-fluorouracil (b) nitrosourea (c) pyrazinamide (d) sulfanilamide

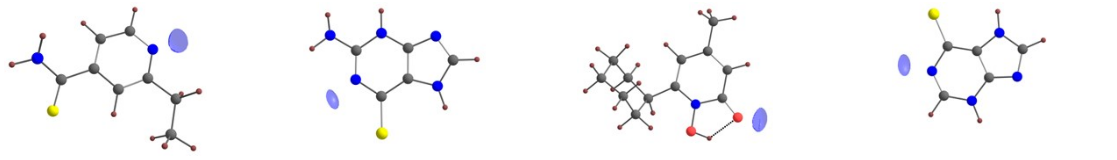

(e) ethionamide (f) 6-thioguanine (g) ciclopirox (h) 6-mercaptopurine

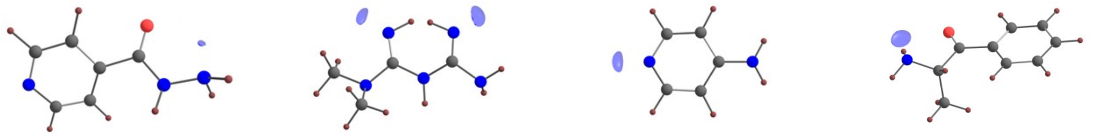

(i) isoniazid (j) metformin (k) 4-aminopyridine (l) cathinone

Figure S1. MESP V_{min} features of (a) 5-fluorouracil, (b) nitrosourea, (c) pyrazinamide, (d) sulfanilamide, (e) ethionamide, (f) 6-thioguanine, (g) ciclopirox, (h) 6-mercaptopurine, (i) isoniazid, (j) metformin, (k) 4-aminopyridine and (l) cathinone. Color code: gray-C, blue-N, yellow-S, maroon-H, cyan-F, red-O.

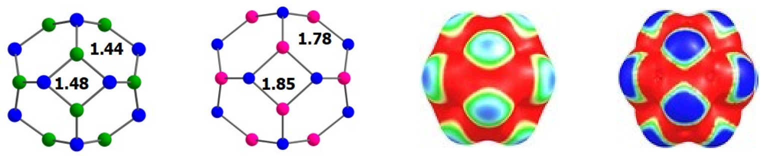

(a) $B_{12}N_{12}$ (b) $Al_{12}N_{12}$ (c) $B_{12}N_{12}$ (d) $Al_{12}N_{12}$

Figure S2. Optimized structures of (a) $B_{12}N_{12}$ and (b) $Al_{12}N_{12}$. [S9] The bond distances are given in Å. Color code: purple-Al, green-B, and blue-N. Also shown are the MESP mapped onto the 0.01 a.u. electron density isosurface of (c) $B_{12}N_{12}$ and (d) $Al_{12}N_{12}$.[S9] Color code: blue -0.04 a.u. to red 0.04 a.u. Blue represents the most electron-rich region and red the most electron-poor region.

Table S2. Change in structural parameters due to drug adsorption on $B_{12}N_{12}$. Bond length in Å and bond angles in degrees.

drug	bond angle of drug		bond length of drug		bond angle of cage	
	drug/$B_{12}N_{12}$	drug	drug/$B_{12}N_{12}$	drug	drug/$B_{12}N_{12}$	cage
5-fluorouracil	124.1	123.4	1.26	1.20	115.9	125
nitrosourea	124.8	125.2	1.25	1.20	115.9	125
pyrazinamide	119.5	116.2	1.33	1.33	117.2	125
sulfanilamide	119.1	120.6	1.45	1.38	117.4	125
ethionamide	120.3	118.3	1.34	1.33	115.8	125
6-thioguanine	106.3	103.2	1.36	1.35	117.1	125
ciclopirox	118.7	116.7	1.30	1.23	115.3	125
6-mercaptopurine	106.3	103.2	1.36	1.35	117.4	125
isoniazid	115.2	119.4	1.41	1.39	117.3	125
metformin	121.3	121.0	1.31	1.27	115.3	125
4-aminopyridine	119.0	115.9	1.34	1.33	115.8	125
cathinone	106.5	108.5	1.48	1.45	116.9	125

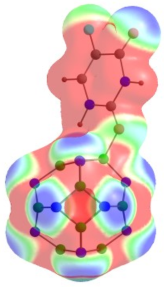
(a) 5-fluorouracil

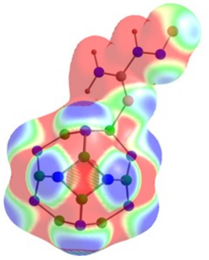
(b) nitrosourea

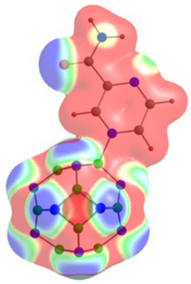
(c) pyrazinamide

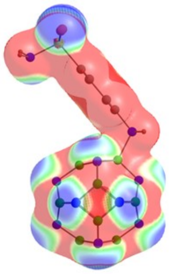
(d) sulfanilamide

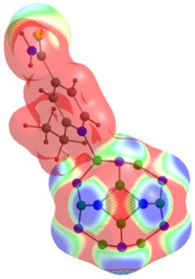
(e) ethionamide

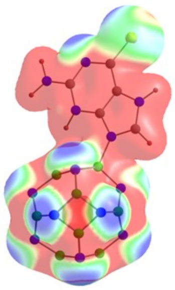
(f) 6-thioguanine

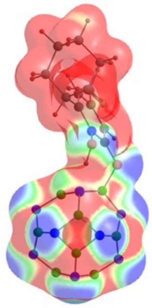
(g) ciclopirox

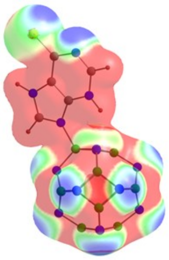
(h) 6-mercaptopurine

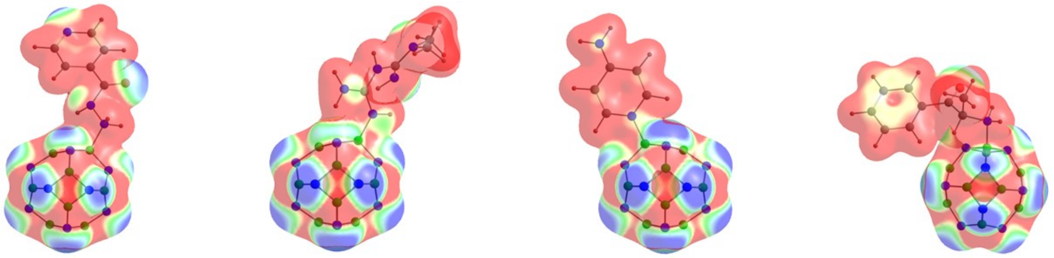

(i)isoniazid (j) metformin (k) 4-aminopyridine (l) cathinone

Figure S3. MESP mapped onto the 0.01 a.u. electron density isosurface of (a) 5-fluorouracil, (b) nitrosourea, (c) pyrazinamide, (d) sulfanilamide, (e) ethionamide, (f) 6-thioguanine, (g) ciclopirox, (h) 6-mercaptopurine, (i) isoniazid, (j) metformin, (k) 4-aminopyridine and (l) cathinone adsorbed on $B_{12}N_{12}$. Color code is same as in Figure S2.

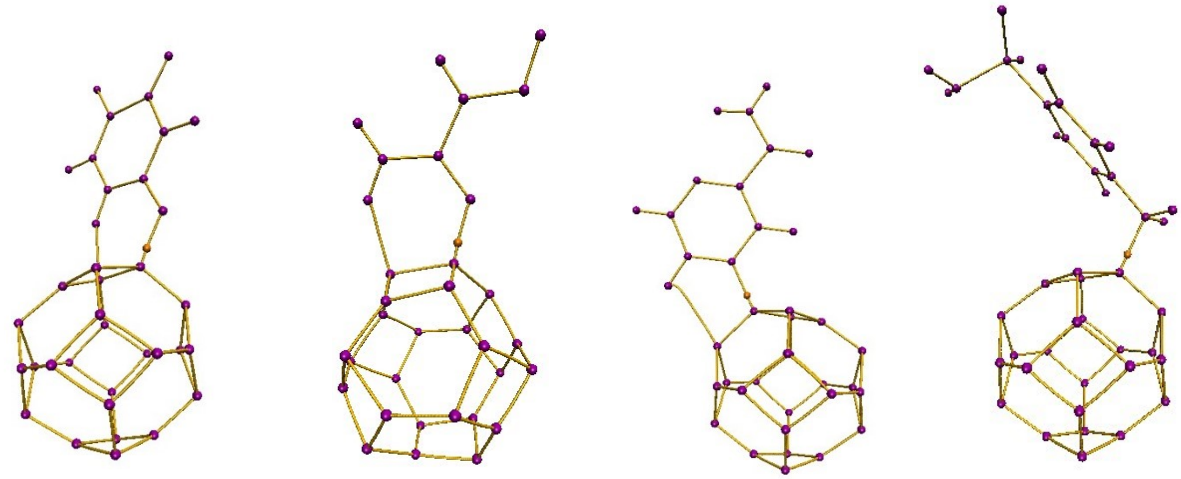

(a) 5-fluorouracil (b) nitrosourea (c) pyrazinamide (d) sulfanilamide

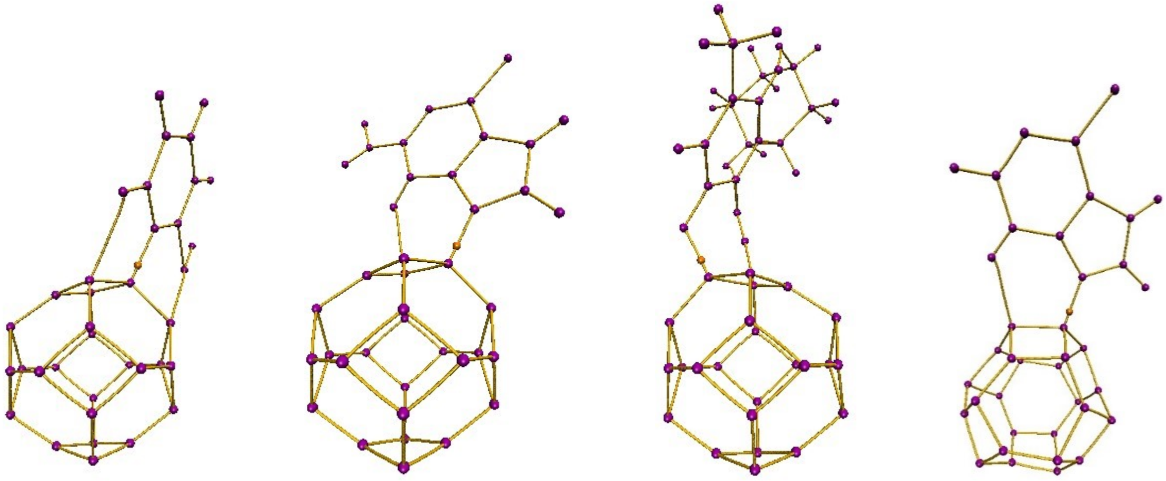

(e)2-mercaptopyridine (f) 6-thioguanine (g) ciclopirox (h) 6-mercaptopurine

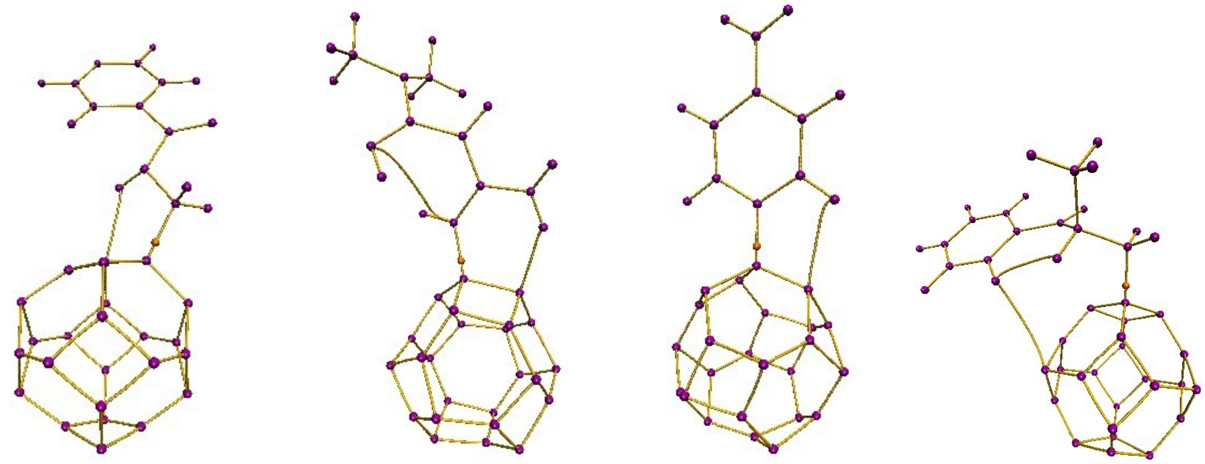

(i) isoniazid (j) metformin (k)4-aminopyridine (l) cathinone

Figure S4. QTAIM plots for (a) 5-fluorouracil, (b) nitrosourea, (c) pyrazinamide, (d) sulfanilamide, (e) ethionamide, (f) 6-thioguanine, (g) ciclopirox, (h) 6-mercaptopurine, (i) isoniazid, (j) metformin, (k) 4-aminopyridine and (l) cathinone adsorbed on $B_{12}N_{12}$.

Table S3. Change in structural parameters due to drug adsorption on $Al_{12}N_{12}$. Bond length in Å and bond angles in degrees.

drug	bond angle of drug		bond length of drug		bond angle of cage	
	drug/$Al_{12}N_{12}$	drug	drug/$Al_{12}N_{12}$	drug	drug/$Al_{12}N_{12}$	cage
5-fluorouracil	125.8	123.4	1.28	1.20	112.9	125
nitrosourea	122.9	125.2	1.26	1.19	113.9	125
pyrazinamide	117.7	116.2	1.26	1.21	118.1	125
sulfanilamide	104.6	107.5	1.51	1.45	118.0	125
ethionamide	120.4	118.3	1.35	1.33	119.6	125
6-thioguanine	105.0	106.9	1.34	1.35	119.9	125
ciclopirox	120.2	113.3	1.35	1.37	117.9	125
6-mercaptopurine	116.1	113.1	1.34	1.32	115.7	125
isoniazid	115.8	119.4	1.42	1.39	121.0	125
metformin	120.9	121.0	1.31	1.27	117.5	125
4-aminopyridine	118.6	115.9	1.34	1.33	118.4	125
cathinone	106.7	108.5	1.49	1.45	120.7	125

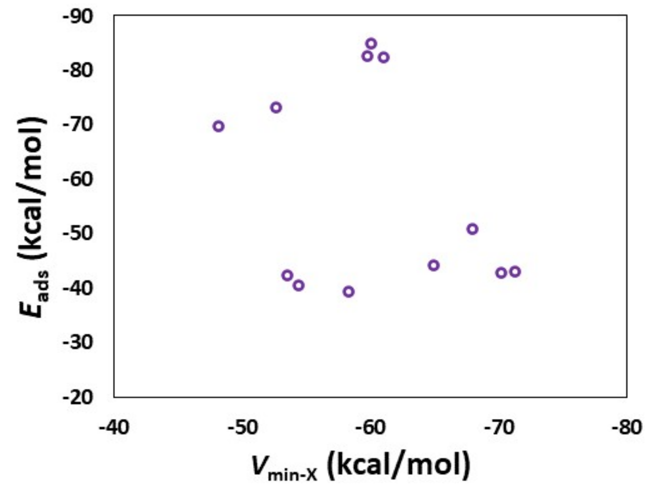

Figure S5. Correlation between V_{min-X} and E_{ads} for drug-adsorbed $Al_{12}N_{12}$ nanocage.

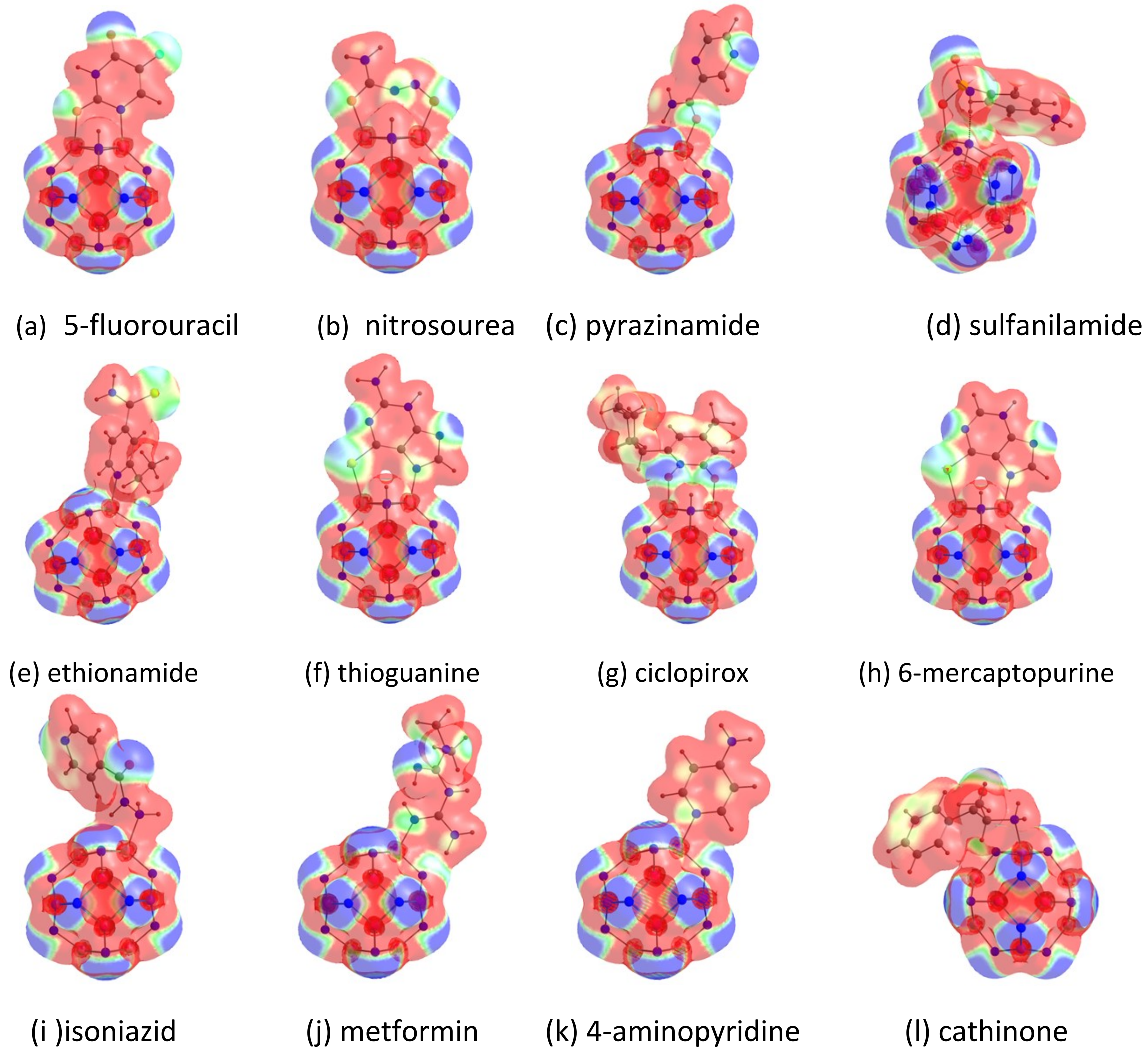

Figure S6. MESP mapped onto the 0.01 a.u. electron density isosurface of (a) 5-fluorouracil, (b) nitrosourea, (c) pyrazinamide, (d) sulfanilamide, (e) ethionamide, (f) 6-thioguanine, (g) ciclopirox, (h) 6-mercaptopurine, (i) isoniazid, (j) metformin, (k) 4-aminopyridine and (l) cathinone adsorbed on $Al_{12}N_{12}$. Color code is same as in Figure S2.

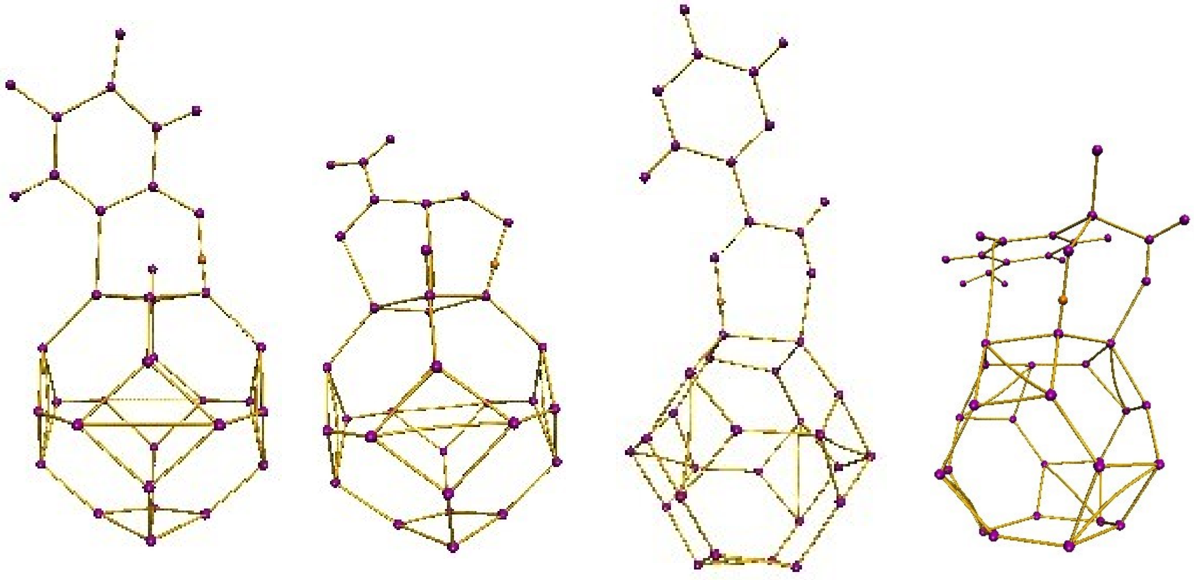

(a) 5-fluorouracil (b) nitrosourea (c) pyrazinamide (d) sulfanilamide

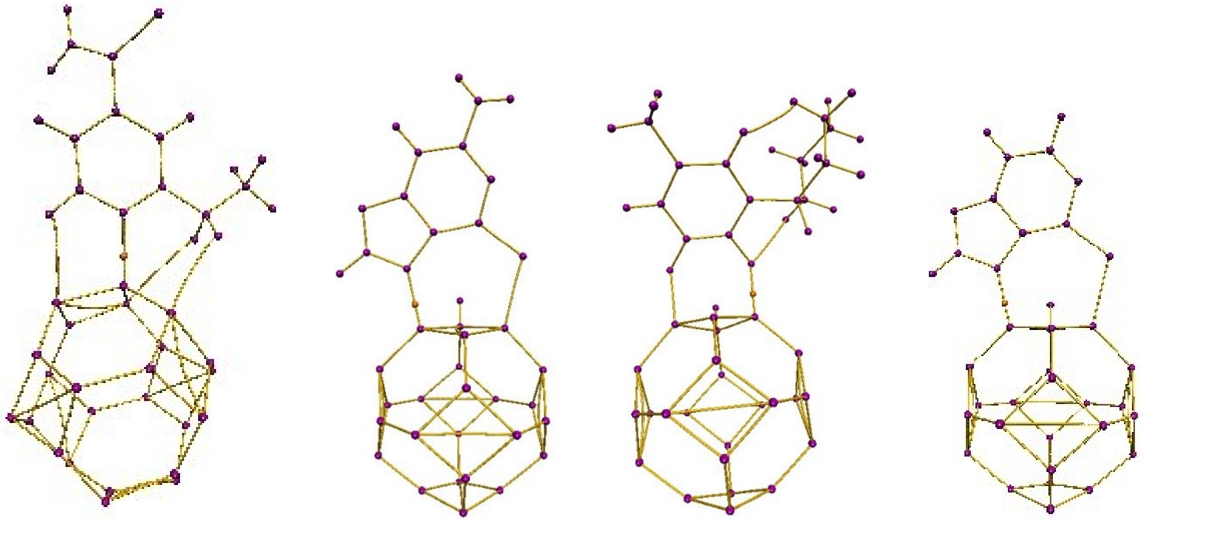

(e) ethionamide (f) 6-thioguanine (g) ciclopirox (h) 6-mercaptopurine

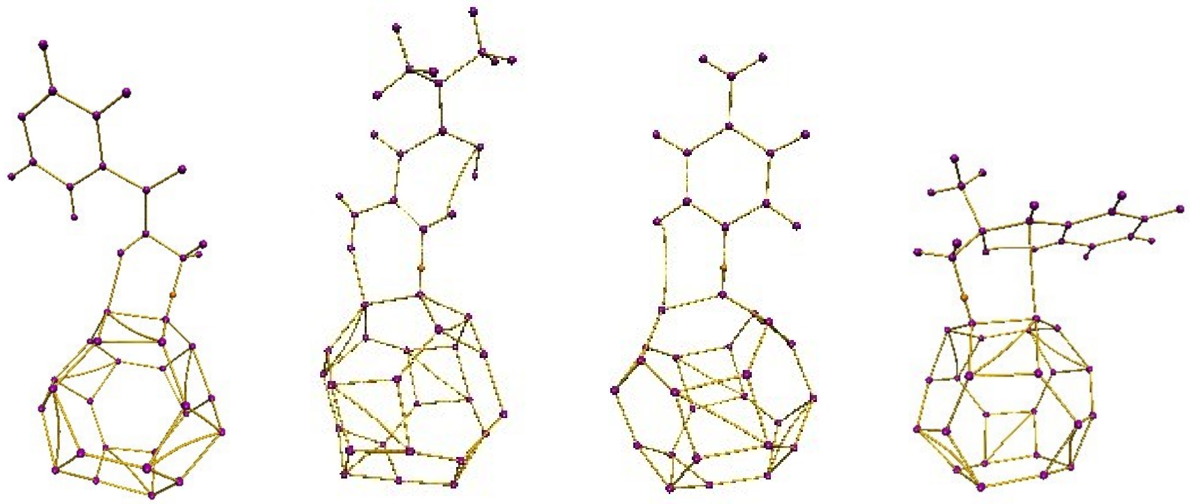

(i) isoniazid (j) metformin (k) 4-aminopyridine (l) cathinone

Figure S7. QTAIM plots of drugs (a) 5-fluorouracil, (b) nitrosourea, (c) pyrazinamide, (d) sulfanilamide, (e) ethionamide, (f) 6-thioguanine, (g) ciclopirox, (h) 6-mercaptopurine, (i) isoniazid, (j) metformin, (k) 4-aminopyridine and (l) cathinone adsorbed on $Al_{12}N_{12}$.

Table S4. Recovery time (τ, in ns) of the drug adsorbed nanocages.

Drug	τ	
	drug/$B_{12}N_{12}$	drug/$Al_{12}N_{12}$
5-fluorouracil	1×10^{5}	5×10^{38}
nitrosourea	8×10^{3}	2×10^{41}
pyrazinamide	1×10^{8}	6×10^{18}
sulfanilamide	3×10^{5}	3×10^{17}
ethionamide	2×10^{11}	4×10^{16}
6-thioguanine	3×10^{12}	1×10^{48}
ciclopirox	3×10^{9}	6×10^{49}
6-mercaptopurine	7×10^{10}	9×10^{47}
isoniazid	7×10^{13}	1×10^{20}
metformin	3×10^{17}	9×10^{24}
4-aminopyridine	5×10^{15}	1×10^{19}
cathinone	1×10^{15}	2×10^{19}

References

S1. F. Azarakhshi, S. Shahab, S. Kaviani and M. Sheikhi, *Lett. Org. Chem.*, 2021, **18**, 640-655.

S2. M. Noormohammadbeigi, S. Kamalinahad, F. Izadi, M. Adimi and A. Ghasemkhani, *Mat. Res. Exp.*, 2019, **6**, 1250g1252.

S3. S. Kaviani, S. Shahab, M. Sheikhi, V. Potkin and H. Zhou, *Comput. Theor. Chem.*, 2021, **1201**, 113246.

S4. R. Padash, A. Sobhani-Nasab, M. Rahimi-Nasrabadi, M. Mirmotahari, H. Ehrlich, A. S. Rad and M. Peyravi, *App. Phys. A*, 2018, **124**, 582.

S5. M. J. Saadh, T. E. Sánchez Herrera, A. Mohammed Dhiaa, O. Villacrés Cáceres, L. M. Flores Fiallos, B. S. Rojas Oviedo, A. A. Omran, M. N. Hawas and A. Elawady, *Mol. Phys.*, 2024, e2314132.

S6. A. S. Ghasemi, M. R. Taghartapeh, A. Soltani and P. J. Mahon, *J. Mol. Liq.*, 2019, **275**, 955-967.

S7. H. R. A. El-Mageed, F. M. Mustafa and M. K. Abdel-Latif, *J. Biomol. Struct. Dyn.*, 2022, **40**, 226-235.

S8. S. A. Aslanzadeh, *Mol. Phys.*, 2019, **117**, 531-538.

S9. R. Geetha Sadasivan Nair, A. K. Narayanan Nair and S. Sun, *Energy Fuels*, 2023, **37**, 14053-14063.
